# 

**Published:** 1877-07

**Authors:** 


### AMERICAN DENTAL ASSOCIATION.

This body meets in Chicago on the first Tuesday of August, and the present indications are that it will be the largest meeting of this Association, that has been held for several years.

We infer this, first, because it is to be held in Chicago. Second, the profession there are making every preparation, to make the meeting a grand success. By reference to the notices in this No. of the Register it will be seen that the most ample provision has been made. And third, our correspondence promises, that almost everybody is going. We hope every member will be present, and all others who can, and let every one come resolved and prepared to add to the work and the interest of the occasion. It is important that during these sessions every member be and remain in his seat, and attend to work during work hours, and when recess comes, then take as much play, fun and sight-seeing as possible. Those who have not seen Chicago for several years, should make arrangements to stay a week and see it.

---

The Annual Meeting of the American Dental Convention and the Dental Association of Maryland and District of Columbia, and a special meeting of the Southern Dental Association, will be held at Deer Park and Oakland, Garrett County, Md., commencing Tuesday, Aug. 14th, 1877, at 11 A. M., and continue through the week.

This is to be a united, but not a consolidated meeting of the three Associations, for while it will consist of a series of joint sessions, each body will control its own internal affairs, and each individual will preserve his distinctive membership in his own body, and at his option acquire membership in the others.

The sessions will be held in the Memorial Church in Oakland, and members will have conveyance at suitable hours between the two places.

This united meeting of the three Associations were arranged with the view of bringing together in social and professional intercourse, to as great an extent as possible, members of the profession from all parts of the country, believing that the best interests of all, both individually and collectively would be thereby promoted. Every effort will be made to have this occasion a pleasant one.

We will have the valuable voluntary co-operation of able and learned members of the Medical Profession; papers will be read from eminent members of the three Associations; our clinical operations will be among the best in the profession—a wider range than usual will be given to the mechanical processes, the battery and the spectroscope will be fully and ably treated in their relations to Dentistry, and if possible the effect of anæthetics on living organisms will be exhibited.

Deer Park and Oakland, six miles apart, are situated on the backbone of the Alleghany Mountains, immediately on the Baltimore and Ohio Railroad. By an arrangement with that Company members of the Associations and those desiring to become members will purchase at regular rates, tickets to Deer Park or Oakland from the following or intermediate points, viz: Washington, Baltimore, Pittsburg, Wheeling, Sandusky, Monroeville, Auburn, Chicago, Cincinnati, Columbus, Portsmouth, Parkersburg, Staunton, Lynchburg and Danville. Returning they will receive certificates at Deer Park or Oakland which will pass them over the route they came to the points at which they purchased their tickets.

The Ohio and Mississippi Railroad will sell to duly accredited members round trip tickets as follows: Louisville to Cincinnati, $5.00, St. Louis to Cincinnati. $12.00. It is believed that other lines connecting with the Baltimore and Ohio, will make for this occasion large reduction in the rates of travel.

The hotels at Deer Park and Oakland can accommodate all who may attend, and the rates will be reduced from $3.50 to $2.50 per day.

The scenery on this road is grand beyond description, and the mountain air is cool, pure and bracing.

It is earnestly hoped that every member of the Associations will attend, and a cordial invitation to be present is extended to all other dentists, to members of the Medical Profession and all others interested in dental affairs.

W. H. Atkinson, *Pres. A. D. C.*
S. J. Cobb, *Pres. S. D. A.*
R. Finley Hunt, *Pres. D. A. Md. & D. C.*

### AMERICAN DENTAL ASSOCIATION.

---

CHICAGO, *July 7th*, 1877.

*Editor of the Dental Register:*

DEAR SIR:—The Seventh Annual Meeting of the American Dental Association, will convene in Chicago, on Tuesday, August 7th, 1877, at the Grand Pacific Hotel.

Arrangements having been made with the proprietors of the above hotel, by the Executive Committe, for hall and all committee rooms in connection therewith, they also have made special rates for the delegates attending the Convention. The regular prices of the hotel are from three to five dollars per day, according to location of rooms, but the rates for delegates have been reduced to $2.50 per day for rooms without baths, and $3.00 per day for rooms with baths. Please notify the proprietors of the hotel, Messrs Jno. B. Drake & Co., by telegram or letter, at as early a date as possible, stating kind of room you wish, so that they can be in readiness for you upon your arrival.

The assembly and Committee Rooms for the use of the American Dental Association have been secured in the Grand Pacific Hotel. The Hotels named below offer reduced rates to the members, as follows;

Palmer House, regular rates, $3.00 to $5.00. Special rates, $2.50 to $3.00.*

Grand Pacific, regular rates, $3 00 to $5.00. Special rates, $2.50 to $3.00.*

Tremont House, regular rates, $3.00 to $4.50. Special rates, $2.00 to $2.50.*

Sherman House, regular rates, $3.00 to $4.50. Special rates, $2.50 to $3.00.*

Clifton House, regular rates, $2.50. Special rates, $2.00.

Mattison House, regular rates, $2.50. Special rates, $2 00 to $2.50.*

Prices to which the star is affixed includes bath rooms. In all other cases, rooms with baths fifty cents extra.

M. S. DEAN,

*Chairman Local Com. Arrangements.*

### CONTENTS.

### COMMUNICATIONS.

### THE DENTAL REGISTER,

A MONTHLY MAGAZINE DEVOTED TO THE INTERESTS OF

**THE DENTAL PROFESSION.**

*TERMS REDUCED TO $2.50 PER YEAR. SINGLE COPIES 25C.*

EDITED BY PROF. J. TAFT, D.D.S.

AND

**Published by SPENCER, CROCKER & CO., Ohio Dental Depot.**

The volume commences in January and closes in December.

The REGISTER being issued monthly is always fresh, therefore Indispensable to the practitioner who desires to keep posted up with the new inventions and improvements which are daily developed. Neither expense nor effort will be spared to give value and variety to its contents. Articles will be illustrated with well executed engravings whenever they will add to the interest or assist to explanation.

Its extensive circulation and frequency of issue make it one of the BEST DENTAL ADVERTISING MEDIUMS in the United States.

All subscriptions must begin with January or July.

Local or special notices, five lines or less, will be inserted for $3 each insertion; over five lines, 50 cts. per line.

All advertising bills payable in advance, or at furthest, after the issue of the first number containing the advertisement. All transient advertisements to be paid for in advance.

☞CONTRIBUTIONS SOLICITED.

Exclusively Editorial Communications should be addressed to Editor.

The latest number of the REGISTER may be ordered with or without other goods at any time, and all letters should be addressed to

***SPENCER, CROCKER & CO., Ohio Dental Depot, Cincinnati, O.***

